# The Foreign Journals: Pathology, Practical Medicine, and Therapeutics

**Published:** 1840-07

**Authors:** 


### PATHOLOGY, PRACTICAL MEDICINE, AND THERAPEUTICS.

### *Hemicrania and Facial Neuralgia cured by Compression of the Carotid Arteries.* By M. Dufresse.

"A working baker, æt. forty, consulted me on the 18th Nov. 1839, for a pain occupying the right side of the cranium and face, extending to beneath the lower jaw, and apparently seated in the facial nerve. The patient also complained of buzzing noise in the head and of tinnitus in the right ear; he was besides subject to vertigo, especially when employed in kneading, and required to be bled for this every three months. I now bled him to fifteen ounces. The following morning I learned that he had not slept, and had been in pain the whole night; I consequently prescribed an opiate liniment. Finding that no improvement had taken place in the evening I again bled him to eight ounces, under which he nearly fainted. On recovering he told me his pain was as severe as ever, but that while faint he had felt none. This recalled to my mind the cases published by M. Dezeimeris (l'Expérience, t. i., p. 65,) illustrating the effects of compression of the carotids, and I determined to give this plan a trial here. The following were the results:—1. *Compression of the right primitive Carotid for ten seconds.* Instantaneous disappearance of the pain, which returned one minute after removal of the pressure. 2. *Compression of the same vessel for fifteen seconds.* Again the pain disappeared instantaneously from its existing position, but shifted to the posterior part of the skull. This new pain ceased with the removal of the pressure, and two minutes after this the original pain reappeared on the right side. 3. *Graduated compression of the same vessel for thirty seconds.* The pain, as before, moved to the posterior left part of the skull. I then pressed on the left carotid, increasing the force here as I diminished that on the right side. The posterior pain disappeared, and no return of either had taken place ten minutes after. The patient slept well and made no complaint of pain in the morning."

*L'Expérience.* No. cxxviii. *Déc.* 1839.

---

### *Statistical Researches on Pneumonia.* By M. Pelletan.

The results in this memoir are drawn from 75 cases of purely inflammatory or bilious and inflammatory pneumonia, observed in M. Bouillaud's wards during the years 1834-5-6, with remarkable exactness and precision. After an orderly arrangement of his cases according to the date of their progress, the author deduces the following propositions:

1. Pneumonia of one lung is more frequent than double pneumonia, in the proportion of 7 to 2. The proportion found by M. Bouillaud was one double pneumonia in 13 cases; that established by M. Andral from 151 cases observed by himself was one in 15, and from 59 cases from various authors one in 7. M. Chomel in 27 fatal cases found nine cases of double pneumonia, that is, one in 3, and in another series of 32 cases he found seven with both lungs inflamed. The exact proportion therefore can be stated only after further researches, which must be conducted with careful regard of the age of the patient and the form of the disease; for it is found that double pneumonia is very much more common in young children and old persons than in adults: and it was more frequent than usual in those who were attacked during the course of influenza.

2. The right lung is more frequently affected than the left in the proportion of 5 to 2. This result accords with the observations of Hales and of Sauvages. M. Chomel also observed the right lung alone affected 28 times, the left only 15; M. Andral found the right alone inflamed 90 times, the left 38; M. Bouillaud, the right 15, the left 8 times; M. Lombard, in 968 cases observed by himself, Andral, and Chomel, found 413 cases of pneumonia confined to the right lung and 260 limited to the left (195 affecting both lungs). M. Gerhard and others have also proved that the difference of liability of the two lungs is still greater in young children.

3. The base of the lung is more disposed to inflammation than its apex in the proportion of one and a half to one. This result also is confirmed by those of other authors, who all (except M. Chomel) have found a similar, though not exactly the same, proportion. M. Andral's results are very nearly the same as M. Pelletan's; M. Bouillaud's present a larger proportion in which the base of the lung was inflamed, and so do those of M. Valleix, which were drawn from the cases of young children.

4. Pneumonia is twice as frequent between the 17th and 37th years of age as at any other period of life. Previous authors differ widely in their expressions on this subject, and almost every period of life has been considered by some as especially liable to pneumonia; it is probable, therefore, that other conditions more powerful than the influence of age have hitherto obscured its real effects.

5. The most general direct cause of pneumonia, in at least seven ninths of the cases, is cold; a fact fully acknowledged by all other authorities under whatever circumstances their observations were made, with the exception of M. Chomel, a large proportion of whose pneumonic patients could assign no cause for their illness.

6. In 13 cases out of 14 the pleura opposite the pneumonic portion of lung was also inflamed, so that there was pleuro-pneumonia. This result is also confirmed by the observations of M. Andral on the pneumonia of adults; but those of MM. Vernois and Valleix show that the coincidence is not so general in very young children.

7. The number of arterial pulsations in the minute affords no exact measure of the degree at which the pneumonia has arrived, or thereby of its severity. M. Chomel agrees on the whole with M. Pelletan on this opinion; but M. Andral and most others ascribe more importance to the indication of the pulse, and regard its great frequency as one of the most alarming signs.

8. The frequency of inspiration is a more serious and important sign for prognosis than the degree of acceleration of the pulse. In all cases, the number of inspirations affords an exact estimate of the degree which the pneumonia has attained.

9. Prostration of strength and delirium are more particularly connected with inflammation of the apex of the lung. Delirium is one of the most serious symptoms, and in all M. Pelletan's cases was followed by death. The first of these assertions is on the whole, though not very distinctly, confirmed by the observations of others; the last accords with the opinion of all, though it is allowed that delirium may occur in the course of pneumonia from circumstances scarcely connected with that disease, and not indicate any peculiar danger.

10. In these 75 cases there were 21 of bilious pneumonia, of which 16 occurred in the cold months and 5 in the warmer season. It does not appear, therefore, that an elevated temperature has any influence in predisposing to the bilious form of the disease. Temperament seems more important in this respect; for in 20 of these bilious cases 11 were of a bilious or sanguineo-bilious habit. It was remarkable that in 17 of these 20 cases the pneumonia affected the base of the right lung, and in only three that of the left. Purely antiphlogistic treatment was as successful in these as in any other cases. All these cases of *bilious pneumonia* were actively inflammatory, and only differed from the rest in being accompanied by symptoms of affection of the liver, and especially by jaundice.

The second part of the memoir relates to the effects of the curative means employed. All the cases were treated on M. Bouillaud's plan of repeated bleedings; the amount of blood varying from 10 to 17 palettes (of about six ounces each), were always drawn in the first three or four days after the patient's reception into the hospital. In pneumonia of one lung, of the first and second degrees of severity thus treated, recovery was the general rule and death a rare exception; only two cases in 55 terminating fatally. In double pneumonia similar treatment was followed by recovery in 11 cases out of 16. In all the cases, recovery took place between the fifth and seventh days of treatment, and between the ninth and thirteenth of the duration of the disease. Pneumonia of the third degree of severity was unaffected by this method of treatment; and both the patients on whom it was employed died.

The results of M. Louis's practice, as well as that of MM. Rilliet and Barthez among children, do not appear so favorable to this plan of bleeding, *coup sur coup;* but its value is entirely confirmed by M. Husson's cases, in which of 43 pneumonic patients bled from once to eleven times each, only three died, and these had the disease under very unfavorable circumstances.

In reference to the duration of the disease also the bleeding system appears equally advantageous; in these cases it continued from nine to thirteen days, while in 50 of M. Louis's, treated with less activity, the average duration was fifteen days; and twenty days in those who were bled between the fifth and ninth day of the affection.

Of blisters, M. Pelletan says that when employed after sufficient abstraction of blood, they never increased the fever, but appeared to have advantageous results; an observation which is on the whole confirmed by the opinions of other good authorities, from which it is deducible that counter-irritation is frequently beneficial in pneumonia affecting adults, always useful in that of old people, and sometimes so in that of children.

*Bulletin de l'Académie Royale de Médecine. Janvier* 31, 1840.

(*Abstract of the Report by M. Rayer.*)

